# Migration of PIP_2_ lipids on voltage-gated potassium channel surface influences channel deactivation

**DOI:** 10.1038/srep15079

**Published:** 2015-10-15

**Authors:** Liping Chen, Qiansen Zhang, Yunguang Qiu, Zanyuan Li, Zhuxi Chen, Hualiang Jiang, Yang Li, Huaiyu Yang

**Affiliations:** 1 State Key Laboratory of Drug Research and Key Laboratory of Receptor Research, Shanghai Institute of Materia Medical, Chinese Academy of Sciences, Shanghai, 201203, China

## Abstract

Published studies of lipid-protein interactions have mainly focused on lipid binding to an individual site of the protein. Here, we show that a lipid can migrate between different binding sites in a protein and this migration modulates protein function. Voltage-gated potassium (Kv) channels have several potential binding sites for phosphatidylinositol-4,5-bisphosphate (PIP_2_). Our molecular dynamics (MD) simulations on the KCNQ2 channel reveal that PIP_2_ preferentially binds to the S4-S5 linker when the channel is in the open state while maintains a certain probability of migrating to the S2-S3 linker. Guided by the MD results, electrophysiological experiments using KCNQ2, KCNQ1, and hERG channels show that the migration of PIP_2_ toward the S2-S3 linker controls the deactivation rate of the channel. The data suggest that PIP_2_ can migrate between different binding sites in Kv channels with significant impacts on channel deactivation, casting new insights into the dynamics and physiological functions of lipid-protein interactions.

Gating is one of the basic properties that define the functions of ion channels^1^. Channel gating generally includes both activation and deactivation processes^2^,^3^. It is believed that the transmembrane S4 and S6 segments, as well as the S4-S5 linker, of voltage-gated potassium (Kv) channels form the main structural determinants of channel activation^4^,^5^,^6^,^7^,^8^,^9^, upon which the outward motion of S4 tightens the S4-S5 linker, perturbing linker-S6-helix packing^9^,^10^,^11^. It has also been well recognized that the minor plasma membrane phospholipid, phosphatidylinositol-4,5-bisphosphate (PIP_2_) can significantly influence activation parameters (e.g. current amplitude and voltage sensitivity) of Kv channels by directly interacting with these structural determinants^12^,^13^. In contrast, despite the growing appreciation on the effects of PIP_2_ and other lipids on channel activation^14^,^15^,^16^, little is known about how lipids regulate the deactivation of ion channels^17^.

The slow deactivation is one of the typical gating properties of the “M current” (e.g. Kv7 or KCNQ channels) and hERG potassium channel, which results in a large “tail” current in response to the repolarization of membrane voltage^18^,^19^. The slow deactivation and large tail currents play an important role in the slow after-hyperpolarization (AHP) associated with action potentials of certain types of neurons and determine the length of Q-T wave of cardiac myocytes^20^,^21^. At present, the molecular mechanism underlying the slow closing process of these channels remains unclear^19^. As in the channel open (activation) process, lipid-channel interactions may also play an important role in the closing (deactivation) process. Test this hypothesis should reveal new functions of lipids in the regulation of ion channel physiology.

Lipid-protein interactions are dynamic because these interactions typically occur on shallow protein surface clefts rather than in deep pockets^22^,^23^,^24^,^25^. However, little is known about the dynamics of lipid–protein interactions despite the recent progresses in identifying potential lipid-binding sites on ion channels. Similar to the studies of ligand-protein interaction, published studies of lipid-channel interactions have mainly focused on lipid binding to an individual site of the channel protein^26^,^27^. Making use of the rich functional and structural results on Kv channels, molecular dynamics (MD) simulations allow visualization of multiple dynamic transitions and large conformational transformation between activated and deactivated states^9^, which when combined with functional approaches, will likely give insightful illumination of the integral influence of PIP_2_ on Kv channels, including how it regulates the slow deactivation process.

Recently, by combining 200-ns MD simulations, site-directed mutagenesis, and electrophysiological tests, we identified PIP_2_ interaction sites on both the S4-S5 linker and S2-S3 linker of the KCNQ2 channel, and found that PIP_2_ affects the activation of KCNQ2 channel differently from that of Shaker and Kv1.2 channels^28^. Here, we performed new and longer (microseconds) MD simulations to examine the dynamics of PIP_2_ interactions with both the open and closed states of the KCNQ2 channel. We observed that although PIP_2_ preferentially binds to the S4-S5 linker in the open state, it exhibits a probability to migrate to the S2-S3 linker, where the lipid resides in the closed state. The migration was abolished by neutralization of the positive charges in the S2-S3 linker, suggesting that PIP_2_ can migrate between different binding sites in Kv channels and the positive charges in the S2-S3 linker are critical for this function. Guided by the MD results, we then performed electrophysiology experiments on wild type (WT) and mutant KCNQ2, KCNQ1 and hERG channels and showed that the migration of PIP_2_ significantly impacts the deactivation kinetics.

## Results

### PIP_2_ migrates between the S2-S3 linker and S4-S5 linker in the open state of KCNQ2

First, we performed long, all-atom, MD simulations on the open state of the KCNQ2 channel. The open-state model was constructed using the crystal structures of Kv1.2 (PDB code 2A79)^29^ and an activated KcsA K^+^ channel (PDB code 3PJS)^30^. Previously, we used this model to successfully identify the binding site of ztz240 (a KCNQ2 activator) and the interaction sites of PIP_2_ in the KCNQ2 channel^28^,^31^. To monitor the dynamics of PIP_2_ interaction with the channel, we placed four PIP_2_ molecules in the inner leaflet of the POPC bilayer far away from the channel. The distance between PIP_2_ and the channel was at least 15 Å (Supplementary Fig. 1a). This system was subjected to two independent 1-μs MD simulations. To increase the statistical power of the simulations, we also built another system, in which the PIP_2_ molecules were located at least 20 Å away from the channel. Two independent 1-μs MD simulations were also conducted on this system (Supplementary Fig. 1b).

**Figure 1:**
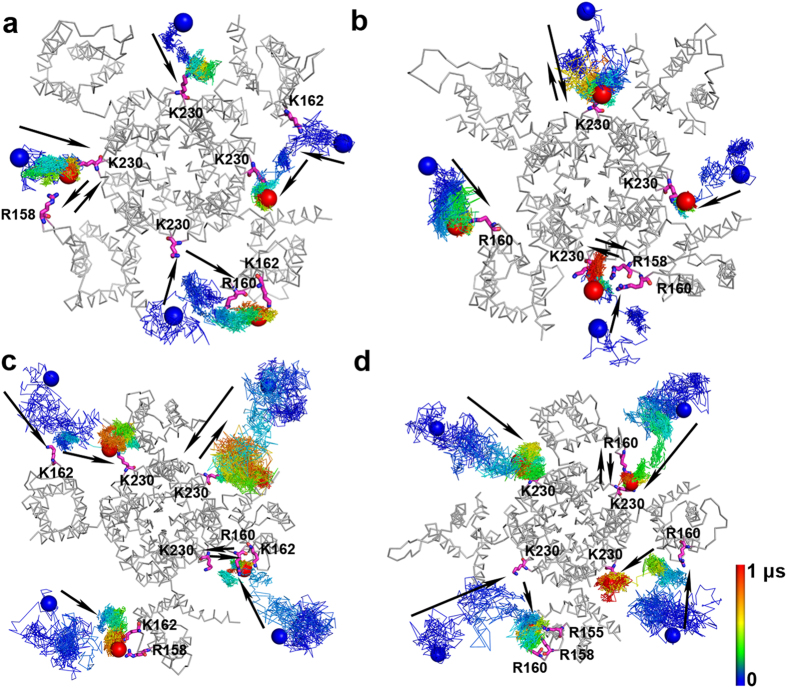
Trajectories of PIP_2_ molecules in the simulations of the open state KCNQ2 channel. (**a**,**b**) Simulations where the initial distance between PIP_2_ molecules and the channel was at least 15 Å. (**c,d**) Simulations where the initial distance between PIP_2_ molecules and the channel was at least 20 Å. The color lines (color-coded from dark blue to red) show the position distribution of 4′-phosphate of PIP_2_ over the 1-μs simulations at 200 ps intervals. The initial (dark blue) and the final (red) positions of the 4′-phosphate of PIP_2_ molecules are displayed as spheres. The final snapshots of the KCNQ2 channels are shown as ribbons, and the critical residues which interact with PIP_2_ molecules in the simulations are presented as magenta sticks. Black arrows indicate the general trends for the movement of PIP_2_ molecules.

From the four independent MD simulations, we captured the motion trajectories of 16 PIP_2_ molecules in total (Fig. 1), which can be divided into seven types: (1) three PIP_2_ molecules interacted with K230 and eventually stabilized at the S4-S5 linker; (2) two PIP_2_ molecules moved to the S2-S3 linker and directly interacted with residues in the linker; (3) two PIP_2_ molecules moved to the S4-S5 linker initially but then migrated to and stabilized at the S2-S3 linker; (4) in contrast to type 3, three PIP_2_ molecules moved to the S2-S3 linker initially and then migrated to and stabilized at the S4-S5 linker; (5) two PIP_2_ molecules moved to the S4-S5 linker initially and then moved back and forth between the S2-S3 linker and the S4-S5 linker without stabilizing at either site; (6) two PIP_2_ molecules moved to the S2-S3 linker initially and then moved back and forth between the S4-S5 linker and the S2-S3 linker without stabilizing at either site; and (7) two PIP_2_ molecules moved to the S4-S5 linker and interacted with K230 initially, but as the simulation time progressed, they gradually detached from this domain and moved to the membrane environment and then moved back and forth between the S4-S5 linker and the membrane environment during the 1-μs simulation time. Statistically, the MD simulations suggested that PIP_2_ preferentially interacts with the S4-S5 linker in the open-state KCNQ2 channel. However, the phospholipid does not always adhere onto the linker, with 9 of the 16 PIP_2_ molecules exhibiting migration motions between the S4-S5 linker and the S2-S3 linker. During these long time MD simulations, there are lots of contacts of PIP_2_ and positively charged residues. Generally, PIP_2_ molecules mainly stayed in three binding models contacted with KCNQ2 channel during the migration between S4-S5 linker and S2-S3 linker: PIP_2_ binding with the S4-S5 linker, PIP_2_ binding simultaneously with the S4-S5 linker and S2-S3 linker, and PIP_2_ binding with the S2-S3 linker (Supplementary Fig. 2).

**Figure 2:**
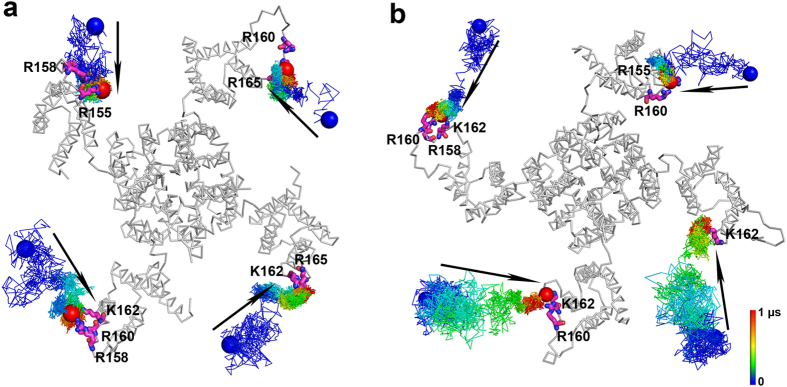
Trajectories of PIP_2_ molecules in the simulations of the closed state KCNQ2 channel. The initial positions of PIP_2_ molecules in each simulation were at least 15 Å (**a**) and 20 Å (**b**) away from the closed KCNQ2 channel. All PIP_2_ molecules moved to the S2-S3 linker in the 1-μs simulations. Display styles are the same as in Fig. 1.

Next, we sought to determine whether PIP_2_ also migrates on the surface of the closed-state KCNQ2 channel. To do this, two closed-state KCNQ2 channel simulation systems were built with the initial positions of PIP_2_ molecules placed similarly to the simulations of the open-state channel. The PIP_2_ molecules were placed in the inner leaflet of the POPC bilayer at least 15 Å or 20 Å away from the channel (Supplementary Fig. 1c,d). The closed-state structure of KCNQ2 was constructed based on the closed-state conformation of the Kv1.2/Kv2.1 chimeric channel reported by Jensen *et al.*^9^ and the crystal structure of a closed KcsA channel (PDB code: 3EFF)^32^. Figure 2 shows the diffusion trajectories of the eight PIP_2_ molecules in the two, independent, 1-μs MD simulations. All PIP_2_ molecules moved to the S2-S3 linker and interacted with positively charged residues, such as R158, R160 or K162. None of the PIP_2_ molecules departed from the S2-S3 linker once it formed the interaction with the linker. These MD results indicate that in the closed state KCNQ2 channel, not only does PIP_2_ favor binding to the S2-S3 linker, but also the lipid does not migrate on the channel surface.

### Positively charged residues in the S2-S3 linker modulate the migration of PIP_2_

Generally, positively charged residues are responsible for PIP_2_ binding to the channel proteins. Therefore, positive charges at the S4-S5 linker and S2-S3 linker should be involved in the interactions with PIP_2_ and its migration between these two domains in the open-state channel. To test this idea, we performed another 1-μs simulation on the open state of a mutated KCNQ2 channel, in which all of the positively charged residues in the S2-S3 linker (R153, R155, R158, R160, K162, R165, and K166) were neutralized by substitutions with an alanine (A) (Supplementary Fig. 1e). In this simulation, all PIP_2_ molecules moved towards positions near K230 in the S4-S5 linker and formed interactions with the nearby residues. None of them moved to the S2-S3 linker or migrated between the S4-S5 linker and the S2-S3 linker (Supplementary Fig. 3). Therefore, positively charged residues of the S2-S3 linker are indeed critical for the migration of PIP_2_ from the S4-S5 linker to the S2-S3 linker in the open state channel. However, mutagenizing all positively charged residues to alanines could disrupt the normal function of the channel. To minimize this possibility, we next studied whether a single mutation influences the migration of PIP_2_.

**Figure 3:**
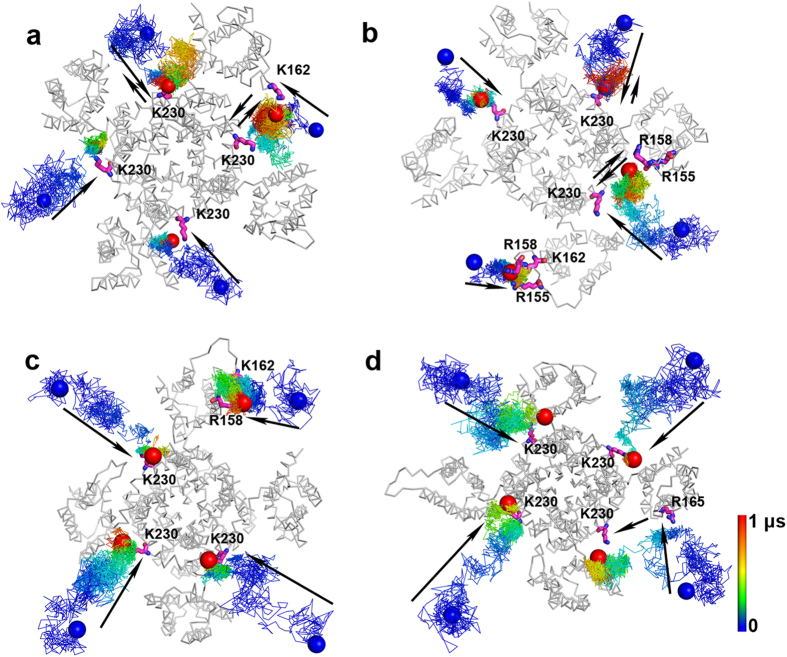
Trajectories of PIP_2_ molecules in the simulations of the open state KCNQ2 channel with the R160A mutation. (**a**,**b**) Simulations where the initial distances between PIP_2_ molecules and the channel were at least 15 Å. (**c,d**) Simulations where the initial distances between PIP_2_ molecules and the channel were at least 20 Å. Display styles are the same as in Fig. 1.

MD simulations on the WT channel suggested that PIP_2_ may frequently interact with R160 on the S2-S3 linker. We thus studied the interactions of PIP_2_ with the R160A mutant of the KCNQ2 channel. Similar to the simulations performed on the WT channel shown in Fig. 1, four MD simulations were conducted on the R160A mutant (Supplementary Fig. 1f,g). Compared with the WT channel, more PIP_2_ molecules interacted with K230 and eventually stabilized at the S4-S5 linker (Fig. 3). Consistent with a decreased affinity to PIP_2_ at the S2-S3 linker, the probability of migration for PIP_2_ between the S4-S5 linker and S2-S3 linker is lower in the R160A mutant than the WT channel in these stimulations. Only three out of the sixteen PIP_2_ molecules showed the migration movements in the R160A mutant. Supplementary Table 1 shows the percentage of time during the simulations when PIP_2_ molecules stayed in the membrane, contacted with the S4-S5 linker, contacted with the S2-S3 linker, and simultaneously interacted with the S4-S5 linker and the S2-S3 linker, for the WT KCNQ2 channel and the R160A mutant. These results clearly reveal a quantitative decrease in the migration probability of PIP_2_ to the S2-S3 linker of the R160A mutant.

### Functional validations for the MD predications

Conformational transitions of the S4-S5 linker following membrane potential changes are critical for Kv channel gating^9^. The MD simulations performed above indicated that PIP_2_ interacts with the S4-S5 linker only in the open state of the KCNQ2 channel, which can be important for the stability of the voltage sensor in the open state^28^. As such, the binding of PIP_2_ at the S4-S5 linker may present a structural hindrance to the transition from the open to the closed state and the rate of dissociation of PIP_2_ from this region should influence the process of channel closing, i.e. deactivation. The MD simulation results suggest that PIP_2_ migration from the S4-S5 linker to the S2-S3 linker facilitates its dissociation from the S4-S5 linker. Therefore, it is quite plausible that disrupting PIP_2_ binding at either the S2-S3 linker or the S4-S5 linker should affect PIP_2_ migration and thereby impact the deactivation kinetics of the KCNQ2 channel. For example, neutralizing positively charged residues at the S4-S5 linker, e.g. K230, should weaken PIP_2_ interaction in this region and drive the lipid further towards the S2-S3 linker, which will accelerate channel closing. By contrast, neutralizing positively charged residues at the S2-S3 linker should hamper the dissociation of PIP_2_ from the S4-S5 linker, and thus slow down the closing process. To verify this hypothesis, we made a series of charge substitution mutations in either the S4-S5 linker or the S2-S3 linker of the KCNQ2 channel and compared their deactivation kinetics with the WT channel by whole-cell patch clamp recordings after heterologous expression in CHO cells.

**Figure 4:**
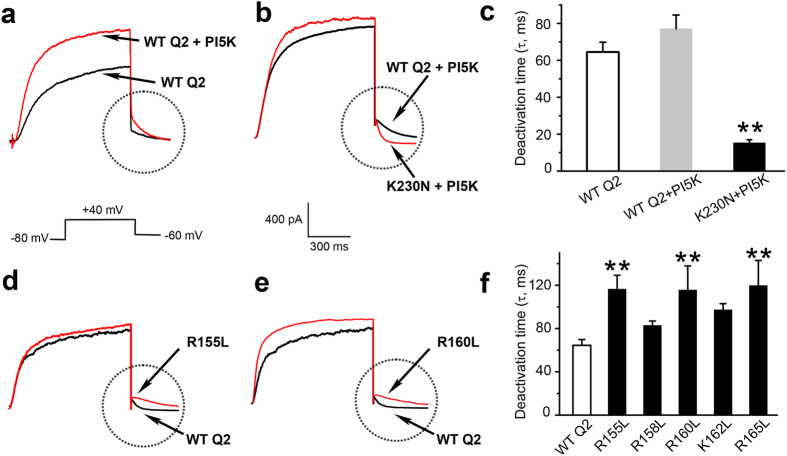
Effect of PIP_2_ on the deactivation process of WT and mutant KCNQ2 channels. (**a**,**b**) Whole-cell currents recorded from CHO cells over-expressing WT and mutant K230N KCNQ2 channels. Currents were elicited by a depolarizing voltage step from the holding potential of −80 mV to 40 mV and then stepping down to −60 mV. The deactivation time constants were obtained by an exponential fit of the tail currents indicated by the circles. PI4(5)-kinase (PI5K) was coexpressed to allow current development of the K230N mutant. (**c**) The statistics of deactivation time constants of the WT KCNQ2 and its K230N mutant. (**d**,**e**) Examples of whole-cell currents overlaid for the WT KCNQ2 and the R155L and R160L mutants. The differences in the deactivation rates are emphasized by circles. (**f**) The statistics of deactivation time constants of the WT KCNQ2 and individual charge neutralizing mutations at the S2-S3 linker as indicated. **P < 0.01, significantly different from the WT by paired *t*-test.

To measure the deactivation kinetics of the KCNQ2 channel, a voltage step from −80 mV to +40 mV was applied to elicit the current. Then a closing voltage step from +40 mV down to −60 mV was applied to allow the development of the tail current, which was fitted by a single exponential equation to obtain the deactivation time constant. Because the K230N mutant displayed very small to nearly no measurable current, we co-expressed PI(4)5-kinase (PI5K) to increase the PIP_2_ concentration in the cell, which, consistent with previous reports^28^,^33^, resulted in enhanced steady-state current amplitude (Supplementary Fig. 4). Under these conditions, the deactivation time constant of K230N (15.3 ± 1.6 ms) was significantly smaller than that of the WT KCNQ2 (77.1 ± 7.4 ms) channel (Fig. 4a–c and Supplementary Table 2). This result supports the MD prediction that neutralizing K230 at the S4-S5 linker should drive PIP_2_ further towards the S2-S3 linker and in turn accelerate channel closing rate.

We then studied the impacts of charge neutralization at the S2-S3 linker on the deactivation kinetics of KCNQ2. We tested R155L, R158L, R160L, K162L, and R165L mutations individually. Although none of the mutations altered the current amplitude (Supplementary Fig. 4), they all showed a tendency to have a slower deactivation process with the deactivation time constants of R155L, R160L, and R165L being significantly slower than that of the WT KCNQ2 channel (Fig. 4d–f and Supplementary Table 2). These results are also consistent with the predictions by the MD simulation, suggesting that K230 on the S4-S5 linker is important for maintaining the closing process, whereas R155, R160, and R165 all contribute to facilitating channel closing by promoting the migration of PIP_2_ to the S2-S3 linker.

### The effect of dynamic PIP_2_ migration on deactivation kinetics of related Kv channels

**Figure 5:**
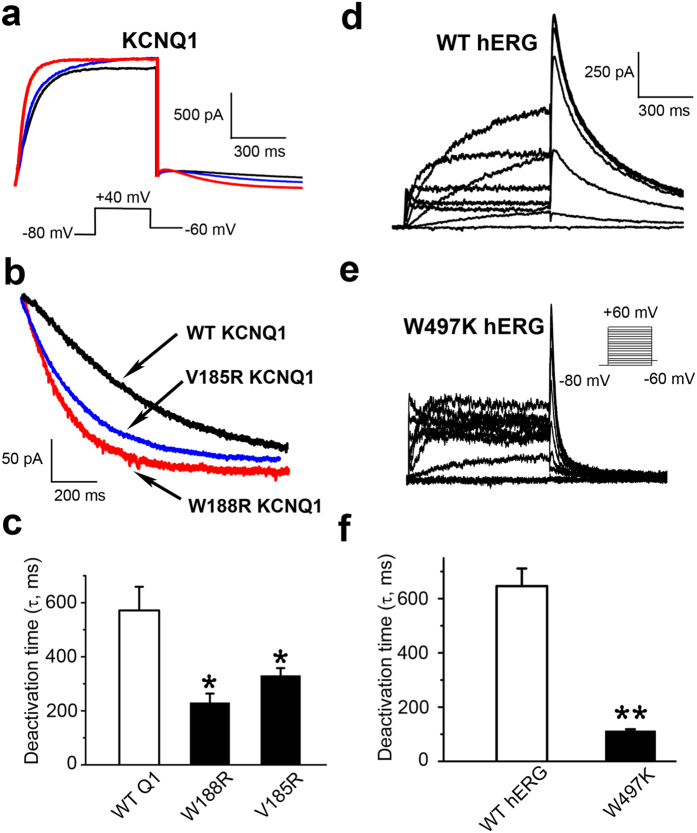
The deactivation time constants of WT and mutant KCNQ1 and hERG channels. (**a**) Representative traces of currents recorded from CHO cells over-expressing WT and mutant KCNQ1 channels. Cells were held at −80 mV, and currents were elicited by a depolarizing voltage step to 40 mV and then stepping down to −60 mV. (**b**) The tail currents were normalized to compare the deactivation process between WT and mutant KCNQ1 channels. (**c**) Introduction of a positive charge to the S2-S3 linker, V185R or W188R, significantly accelerated the closing process of KCNQ1, as indicated by the decreased deactivation time constants. (**d,e**) Whole cell currents were recorded from CHO over-expressing the WT and mutant hERG channels. Cells were held at −80 mV. Voltage steps from −80 mV to +60 mV with 20 mV increments were applied for 800 ms before stepping down to −60 mV. (**f**) The W497K mutation at the S2-S3 linker of the hERG channel significantly accelerates its closing process. The deactivation time constants were initially measured by two-exponential fitting, and then compared by a weighted time constants (see the detail in Online Methods). *P < 0.05; **P < 0.01, significantly different from the WT, by paired t-test.

Having demonstrated the importance of the dynamic migration of PIP_2_ at the cytoplasmic surface of the KCNQ2 channel in the regulation of deactivation, we sought to determine whether this mechanism also operates in other voltage-gated potassium channels. We first tested the KCNQ1 channel, which belongs to the same Kv7 family as the KCNQ2 channel. However, although the S2-S3 linker of KCNQ1 is highly homologous to that of KCNQ2, it contains less number of positively charged residues. In particular, residues corresponding to R155 and R158 of KCNQ2 are neutral in KCNQ1 (V185 and W188, respectively). If the S2-S3 linker of KCNQ1 has a similar role as that of KCNQ2 in modulating channel closing, then mutating V185 and W188 to positively charged residues should increase the closing rate. Not surprisingly, our mutagenesis and electrophysiology experiments showed a significant decrease in the deactivation time constant of either V185R or W188R to about a half of that of the WT KCNQ1 channel (Fig. 5a–c and Supplementary Table 3).

From sequence alignments of the S2-S3 linkers of commonly known Kv channels^28^,^34^, we noticed a clear segregation of two types of Kv channels: one containing 11 amino acid residues (short loop) while the other having 20–24 residues (long loop). We wondered if the long S2-S3 linker channels, including the Kv7 family and the hERG channel, all share a similar mechanism of PIP_2_ regulation on channel deactivation, especially with respect to its dissociation from the S4-S5 linker and migration to a different site. However, mutating the two positively charged residues, R488 and K495, as well as the very weakly positively charged H492, in the S2-S3 linker of the hERG channel individually to an alanine (A) did not significantly alter the deactivation time constant (Supplementary Fig. 5 and Supplementary Table 4), which is already quite large for the WT channel that is characterized by a very slow closing process^35^. This was not too surprising given that there are only two positively charged residues in the S2-S3 linker of the hERG channel, compared with as many as seven positively charged ones in the corresponding loop of KCNQ2. Therefore, the very slow deactivation of hERG may be due to insufficient positive charges at the S2-S3 linker to entice PIP_2_ away from the S4-S5 linker, where the dissociation rate of the lipid determines how fast the channel can close. If this is true, then introducing new positive charges in the S2-S3 linker may facilitate the dissociation of PIP_2_ from the S4-S5 linker and accelerate the rate of channel closing. To test this possibility, we made mutations at the S2-S3 linker with the goal to increase the collective positive charge in this region. We found that mutation Y493K displayed no function, while the functional mutation W497K in the S2-S3 linker, dramatically decreased the deactivation time constant of hERG by nearly 5 fold, from 630 ± 57 ms to only 135 ± 15 ms (Fig. 5d–f and Supplementary Table 4), turning the very slowly closing hERG channel into a “normal” channel. The mutation W497K retained other characteristics of the hERG channel, such as a faster inactivation than activation and a faster recovery from inactivation than deactivation. These results support the idea that the hERG channel shares a similar mechanism of PIP_2_-dependent regulation of deactivation as the KCNQ channels, involving PIP_2_ dissociation from the S4-S5 linker and migration to the S2-S3 linker.

## Discussion

The “M current” is a sub-threshold voltage-gated K^+^ current that is encoded by Kv7/KCNQ channels and is characterized by slow deactivation and non-inactivation, which are critical for stabilizing the membrane potential and regulating the firing rate of excitable cells^36^,^37^,^38^. The KCNQ1 channel plays an important role in cardiac function, and the KCNQ2 channel is a target of anti-epilepsy therapeutics^39^,^40^. The hERG and KCNQ1 channels form the major repolarizing current (IKr) in ventricular myocytes, which helps maintain the QT interval^41^,^42^. The slow kinetics of the hERG channel closure is thought to be important for membrane potential repolarization of ventricular cells and prevention of premature beats^41^,^43^,^44^. However, the molecular mechanism underlying the slow deactivation of neither KCNQ nor hERG channel has been clearly elucidated. The present study strongly suggests that the deactivation is regulated by the dynamic migrations of PIP_2_ among its multiple binding sites in both of these channel types.

The regulatory function of PIP_2_ on KCNQ channels and hERG channels has been extensively investigated. Previous reports have demonstrated the importance of PIP_2_ in channel activation and the mechanism by which receptors inhibit these Kv channels through hydrolyzing PIP_2_^45^,^46^. Recently, the direct interaction of PIP_2_ with the S4-S5 linker of Kv channels received much attention, because of the implication that PIP_2_ may be a necessary cofactor for the voltage-dependent gating of Kv channels^12^,^13^,^28^. Further evidences confirmed that PIP_2_ played a vital role in the coupling movement of voltage sensor and pore domain, and PIP_2_ binding sites on KCNQ1 channel included the positively charged residues of S4-S5 linker, bottom of S4 and S2-S3 linker, especially at the closed and rest state of KCNQ1 channel^47^,^48^. These evidences support that shift of PIP_2_ binding sites complies with the conformational transition of coupling movement during gating process. Early evidences indicate that a cluster of cytoplasmic positive charged residues close to membrane interface could form PIP_2_ binding sites to affect the activation of KCNQ channels^49^. Although currently there are still lacking appropriate structural information of cytoplasmic domain of KCNQ channels, we found the mutation R325A at proximal S6 segment displayed faster deactivation speed, similar to the effect of K230N at S4-S5 linker (Supplementary Fig. 6). The small current amplitude and faster closing speed of these mutations (R325A, K230N) imply that hindrance of PIP_2_’s binding to its activation sites will favor PIP_2_’s migrating to the S2-S3 linker (closing binding sites), resulting an acceleration of deactivation process. Upon membrane depolarization, the S4-S5 linker moves to its position in the open-state conformation, where its interaction with PIP_2_ can increase the stability of the entire complex^28^. However, this interaction may also present a hindrance on the return of the S4-S5 linker upon repolarization to its position in the closed-state conformation. In such a case, the dissociation of PIP_2_ from the S4-S5 linker would be rate-limiting to channel deactivation. Presumably, the common principles guiding lipid binding and dissociating, such as a direct and reversible binding-dissociation process of lipid-protein interaction, may apply to the binding dynamics of PIP_2_ in the channel. However, such a simple mechanism lacks the versatility for Kv channels to adapt different needs in regulating repolarization because of the strict dependence on the affinity of PIP_2_ binding to a single site. Our MD simulations indicated that the PIP_2_ interaction with the S4-S5 linker of KCNQ2 is not a simple association-dissociation process. Instead, it is strongly influenced by the nearby S2-S3 linker due to the ability of the lipid to dynamically migrate between the two sites (Fig. 1). This arrangement makes it possible for different Kv channels to adapt diverse functional needs through changes in the affinity to PIP_2_ in either the S4-S5 linker or the S2-S3 linker, as well as the distance between these two sites via evolution and/or post-translational modifications.

Mutations of Kv channels increase the risk of Romano-Ward syndrome (RWS) and long-QT syndrome (LQTs)^50^. Clinical screening has detected more than 40 mutations in the S2-S3 linkers of KCNQ1 and the hERG channel that may be related to RWS and LQTs (http://www.fsm.it/cardmoc). Likewise, mutations in the S2-S3 linker of KCNQ2 lead to a high risk of neurological diseases^51^. On the other hand, the role of the S2-S3 domain in voltage-gated channels has often been neglected during the past decades. Our results reveal that the S2-S3 linker plays an important role in the closing process of Kv channels through modulation of the dissociation of PIP_2_ from the S4-S5 linker. In functional studies, the deactivation rates of KCNQ and hERG channels are correlated with the number of positive charges in the S2-S3 linker, with the rate decreased by reducing the positive charge, but increased by introducing a new positively charged amino acid in this region (Figs 4 and 5). The discovery of this new function for the S2-S3 linker should provide structural and functional insights into the pathophysiology of related diseases. Additional studies on mechanisms that regulate PIP_2_ migration in KCNQ and hERG channels will likely shed new lights on designing drug therapies to treat diseases.

**Figure 6:**
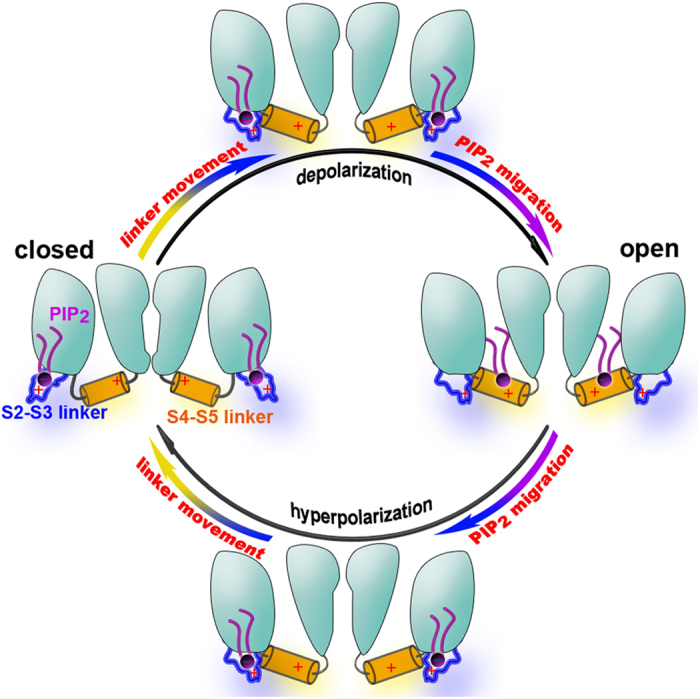
The model of PIP_2_ migration mechanism on the KCNQ2 channel. In the closed-state, PIP_2_ molecules are adsorbed to the S2-S3 linker of KCNQ2 channel. Upon channel activation, the conformation of S4-S5 linker of KCNQ2 channel is changed firstly, and then PIP_2_ migrates from the S2-S3 linker to the S4-S5 linker. After that, PIP_2_ is anchored at the S4-S5 linker, which makes the channel stable at the open state. When the channel is closing, PIP_2_ firstly migrate from the S4-S5 linker to the S2-S3 linker, and then the conformation of S4-S5 linker switches to closed state from open state. PIP_2_ molecules are shown as magenta sticks.

Our results clearly support that the conformational transformation of S4-S5 linker is coupled to the binding and dissociation of PIP_2_. The dynamic process of these coupling is a fundamental question for understanding the gating process of Kv channels. Our simulations and experimental results support a model in Fig. 6. At the closed state, PIP_2_ binds stably to the S2-S3 linker without migration to the S4-S5 linker. The opening of the channel due to the voltage sensor movement brings the S4-S5 linker to its position in the activation (open) conformation, enabling the migration of PIP_2_ from the S2-S3 linker to the S4-S5 linker. The binding of PIP_2_ to this region helps stabilize the open-state conformation and as a result, it also prevents the transition from the open to the closed state upon membrane repolarization that should drive the voltage sensor and the S4-S5 linker back to their original positions in the closed conformation. Therefore, the rate of PIP_2_ dissociation from the S4-S5 linker can determine the closing or deactivation kinetics of the channel. The migration of PIP_2_ to S2-S3 linker helps regulate its dissociation from the S4-S5 linker, which allows a more dynamic control of the deactivation process than simply relying on intrinsic off rate of the lipid to dissociate from the S4-S5 linker. Our functional results are entirely consistent with this model, supporting the idea that PIP_2_ migration between the S4-S5 linker and the S2-S3 linker, and therefore the consequent effect on PIP_2_ dissociation from the S4-S5 linker, can be rate-limiting to deactivation of KCNQ and hERG channels. Therefore, the dynamic migration of PIP_2_ on the cytoplasmic surface plays an important role in the physiological functions of these Kv channels through regulation of their deactivation.

MD simulations have played a critical role in identifying the dynamic migrations of very small molecules in proteins, such as CO migration in myoglobin^52^. Our results suggest that MD simulations can also be applied as a powerful tool to reveal the migration of larger molecules on protein surface. Besides PIP_2_, other membrane lipids also display multiple interactions with membrane proteins. For example, various cholesterol interaction sites have been found in G-protein coupled receptors^27^,^53^. Our current work may provide a good reference for future studies on the lipid regulation of other ion channels and membrane proteins in general.

## Methods

### Structural models of the open- and closed-state KCNQ2 channels

The open-state and closed-state KCNQ2 channel structure models used in molecular dynamic simulations were generated by homology modeling. The crystal structure of Kv1.2 (PDB code 2A79)^29^ provides the information about the open-state conformation. Recently, using long time all-atom MD simulation, Jensen *et al.*^9^ reported the closed-state conformation of a Kv1.2/Kv2.1 “paddle chimera” channel. Based on these structures, we modeled the structures of the transmembrane domains in open- and closed-state KCNQ2 channels. The structures of the cytoplasmic C termini of the open- and closed-state KCNQ2 channels were modeled based on the open- and closed-state KcsA crystal structures (PDB codes 3PJS and 3EFF)^30^,^32^. The N-terminal and the remaining C-terminal extensions of the open- and closed-state KCNQ2 channel were not modeled due to the absence of equivalent proteins as template structures. Multiple sequence alignments of the templates and KCNQ sequences were performed by using the CLUSTALW Web server (www.ebi.ac.uk/Tools/msa/clustalw2), and the highly conserved residues were used to guide the alignment. After manually adjusting the alignments, homology models of the KCNQ2 channel in the open- and closed-state were built with program MODELLER in Discovery Studio 2.6 (Accelrys Software Inc.) based on the templates mentioned above. The generations of these models are described in detail elsewhere^28^. The accuracy of the models had also been analyzed and evaluated by a variety of methods, which all suggested the suitable quality of the open- and closed-state KCNQ2 channel structures^28^. The structural models for mutant KCNQ2 channels used for MD simulations were built from the open-state KCNQ2 model by mutating R160 or all of the basic residues (R153, R155, R158, R160, K162, R165, and K166) located in the S2-S3 linker to alanines.

### Simulation systems

The open- and closed-state WT and R160A mutant KCNQ2 channel models were embedded separately into a palmitoyloleolyl phosphatidylcholine (POPC) bilayer by aligning the protein’s axis of symmetry with the bilayer normal. In each system, lipids located within 1 Å of the KCNQ2 channel were removed, and four PIP_2_ molecules were added manually to the inner leaflet of the POPC bilayer. The initial positions of the PIP_2_ molecules were at least 15 Å or 20 Å away from any atom of the channel, respectively (Supplementary Fig. 1). The simulation systems for KCNQ2 mutants with positive charges neutralized in the S2-S3 linker were also built as described above with the exception that the initial positions of the four PIP_2_ molecules were no less than 15 Å away from the mutant model. Subsequently, each system was solvated by TIP3P waters with 0.15 M KCl. Each simulation system included ~200,000 atoms (140 × 140 × 110 Å).

### Molecular dynamics simulations

All MD simulations were performed using the GROMACS 4.6 package with the lsobaric-lsothermal (NPT) ensemble and the CHARMM36-CAMP force field. Please refer to the previous article for more details of MD-simulation parameters^28^. Energy minimizations were first performed to relieve unfavorable contacts, followed by equilibration steps of 27 ns in total to equilibrate the lipid bilayer and the solvent, with restraints (isotropic force constant κ = 1×10^3^ kJ·mol^−1^·nm^−2^) on PIP_2_ and the main chain of the transmembrane domain. We simultaneously relaxed all of the loops during the equilibration steps, when the PIP_2_ molecules were still distant from the KCNQ2 channel with the minimum distance between PIP_2_ molecules and the KCNQ2 channel no less than 15 Å or 20 Å as was initially set. Subsequently, we performed four independent 1-μs all-atom molecular dynamic simulations of the open-state WT KCNQ2 simulation systems (each system, according to the initial position of the PIP_2_ molecules, has two independent MD simulations). Two independent 1-μs MD simulations of the closed-state KCNQ2 simulation system were also carried out (each system, according to the initial position of the PIP_2_ molecules, has one independent MD simulation). After that, we performed four independent 1-μs all-atom molecular dynamic simulations of the open-state R160A mutant simulation systems (each system, according to the initial position of the PIP_2_ molecules, has two independent MD simulations, as for the open-state WT KCNQ2 simulations described above). Furthermore, we performed an additional 1-μs MD simulation for the KCNQ2 mutant model in which all positive charges in the S2-S3 linker were neutralized. As the KCNQ2 channel contains a large cytoplasmic domain (536 residues) after residue 337, the motions of the C-terminal residues 313–337 should be restrained by the cytoplasmic domain. Since no similar structure is available in the databank to allow construction of a homology model for this domain, we applied conformational restraints (isotropic force constant κ = 1×10^3^ kJ·mol^−1^·nm^−2^) to the Cα atoms of residues 313–337 to mimic the effects of the missing cytoplasmic domain on the motion of these residues. Analysis of the trajectories was performed using Gromacs analysis tools. PyMOl (The PyMOL Molecular Graphics System, Version 1.3, Schrödinger, LLC) was used to visualize the structure models and generate figures.

### Cell culture and transfection

CHO cells were used for electrophysiological analysis as described previously^45^. Cells were grown in 100 mm tissue culture dishes (Corning Incorporated) in DMEM/F12 (Gibco) with 10% FBS (Gibco), 100 U/mL penicillin (Cellgro) and 100 μg/mL streptomycin (Cellgro) in a humidified incubator at 37 °C (5% CO_2_). Cells were passaged every 2–3 days. The cells were transiently transfected with the plasmids using PolyJet™ reagent (SignaGen) according to the instructions of the manufacturer. Cells were used 36–96 hours after transfection for electrophysiological experiments. To facilitate identification of transfected cells, cDNA encoding green fluorescent protein (GFP) was cotransfected with the cDNA of the interested channel at a 1:10 ratio. Cells displaying green fluorescence were used for electrophysiological recording.

### Electrophysiological recording

Whole-cell patch clamp recordings were performed at room temperature (22–25^o^ C) with an Axopatch-200B amplifier (Molecular Devices) on transfected CHO cells. The microelectrodes were pulled from Flaming/Brown type micropipette puller (P-97; SUTTER INSTRUMENT) and had the resistances of 3–6 MΩ when filled with a solution containing (in mM): 140 KCl, 2 MgCl_2_, 10 EGTA, 1 CaCl_2_, 10 HEPES (pH set to 7.3 using KOH). Cells were bathed in a solution containing (in mM): 150 NaCl, 5 KCl, 0.5 CaCl_2_, 1.2 MgCl_2_, 10 HEPES (pH set to 7.3 using NaOH). Current signals were low-pass filtered at 1 kHz and digitized at a 10 kHz sampling frequency using DigiData 1440 A. Data were analyzed using pClamp 10.2 software (Molecular Devices).

### Data analysis

The parameters of whole-cell patch clamp recordings of KCNQ2 and KCNQ1 channels were set up as follows: Cells were held at −80 mV and then depolarized to potentials from −80 mV to +80 mV with 17 steps (10 mV increment), each for a duration of 800 ms, and then stepped down to −60 mV. For recording of hERG channel currents, the holding potential was set at −80 mV, and depolarization steps from −80 mV to +60 mV in 10 mV increments, each had a duration of 800 ms and was followed by stepping down to −60 mV, were applied.

To quantify deactivation kinetics, the decaying process of tail currents for hERG channel was fitted by a bi-exponential function as follows:


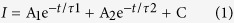


where *I* is the current amplitude, *t* is time, A_1_ and A_2_ and τ_1_ and τ_2_ are the amplitudes and time constants for the slow and fast components, respectively, and C is a constant. To compare the difference between wild type construct and its mutants, the weighted mean of the time constants was calculated as: τ_deact_ = (A_1_τ_1_ + A_2_τ_2_)/(A_1_ + A_2_).

For KCNQ1 and KCNQ2 channels, the decaying portion was fitted using a single exponential function:


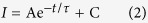


pClamp 10.2 and origin were used to perform Curve fitting and statistical comparisons. Data are shown as the means ± SEM. *n* represented the number of tested cells. Two-sample t-test was used to perform statistical analysis.

## Additional Information

**How to cite this article**: Chen, L. *et al.* Migration of PIP^2^ lipids on voltage-gated potassium channel surface influences channel deactivation. *Sci. Rep.* **5**, 15079; doi: 10.1038/srep15079 (2015).

## Supplementary Material

Supplementary Information
