# Supplementary material for: Migration of PIP2 lipids on voltage-gated potassium channel surface influences channel deactivation
Source: Sci Rep. 2015 Oct 15;5:15079. doi: 10.1038/srep15079 (PMC4606798; doi:10.1038/srep15079)
Supplement: Supplementary Information [file srep15079-s1.doc]

**Supplementary Information**

**Migration of PIP_2_ lipids on voltage-gated potassium channel surface influences channel deactivation**

Liping Chen, Qiansen Zhang, Yunguang Qiu, Zanyuan Li, Zhuxi Chen, Hualiang Jiang, Yang Li, Huaiyu Yang

State Key Laboratory of Drug Research and Key Laboratory of Receptor Research, Shanghai Institute of Materia Medical, Chinese Academy of Sciences, Shanghai, 201203, China.

**Supplementary Figures**

**
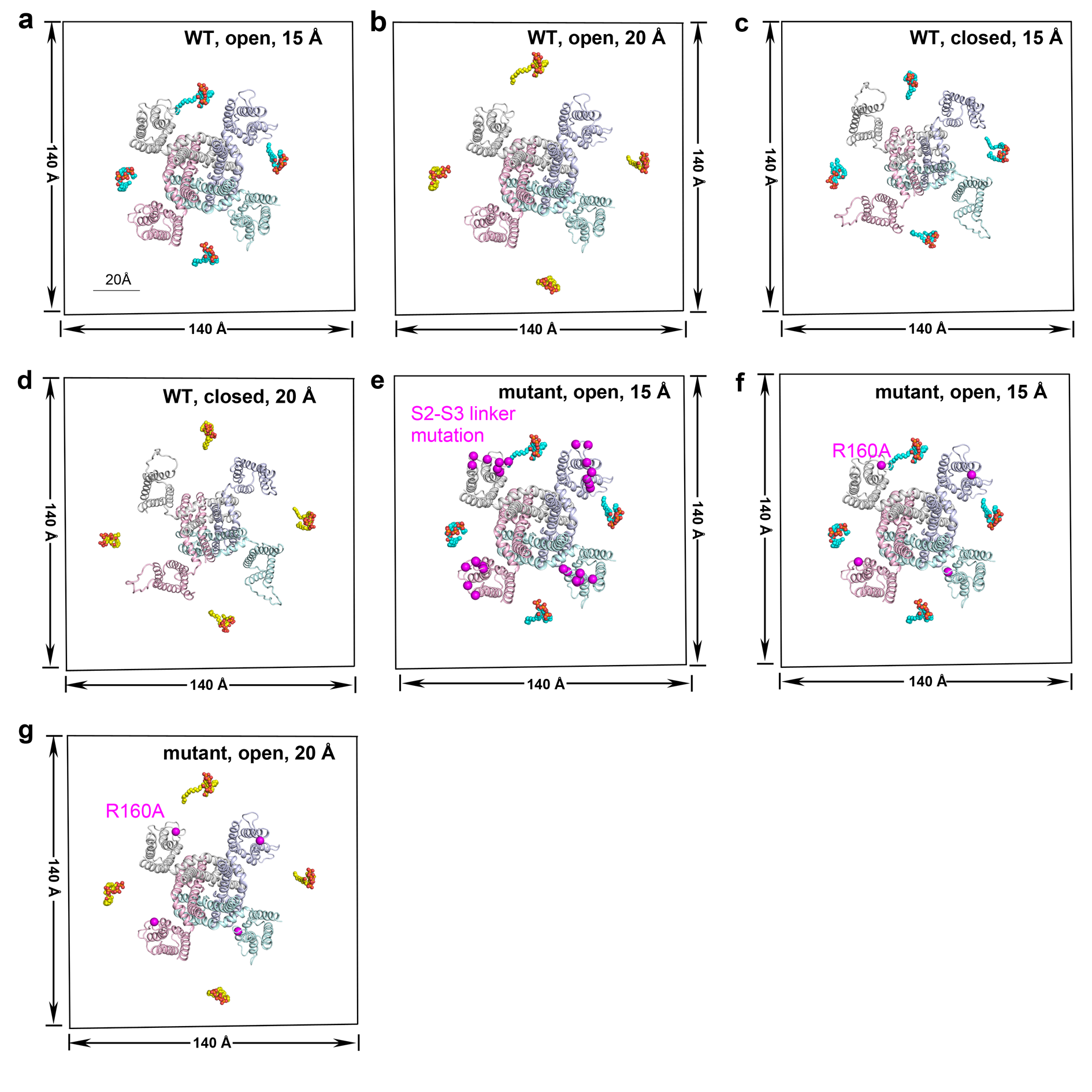
**

**Supplementary Figure 1.** Simulations systems of the open state (**a**, **b**, **e**-**g**) and closed state (**c**, **d**) of the WT (**a**-**d**) and mutant (**e**-**g**) KCNQ2 channels. The channels are shown in cartoon, viewed from the intracellular side. PIP_2_ molecules are shown in spheres. The PIP_2_ molecules shown in cyan spheres (**a**, **c**, **e**, **f**) are at least 15 Å far from the channel and the PIP_2_ molecules shown in yellow spheres (**b**, **d**, **g**) are at least 20 Å far from the channel. In the S2-S3 linker mutation system with several mutations (**e**), all of the basic residues (R153, R155, R158, R160, K162, R165, and K166) in the S2-S3 linker are mutated into alanine. In the simulation systems of R160A mutant KCNQ2 (**f** and **g**), R160 was mutated into alanine. Magenta balls in **e**-**g** indicate the mutant sites. For clarity, POPC lipids, water, and ions are not shown. The size of each simulation system was scaled and labeled.

**
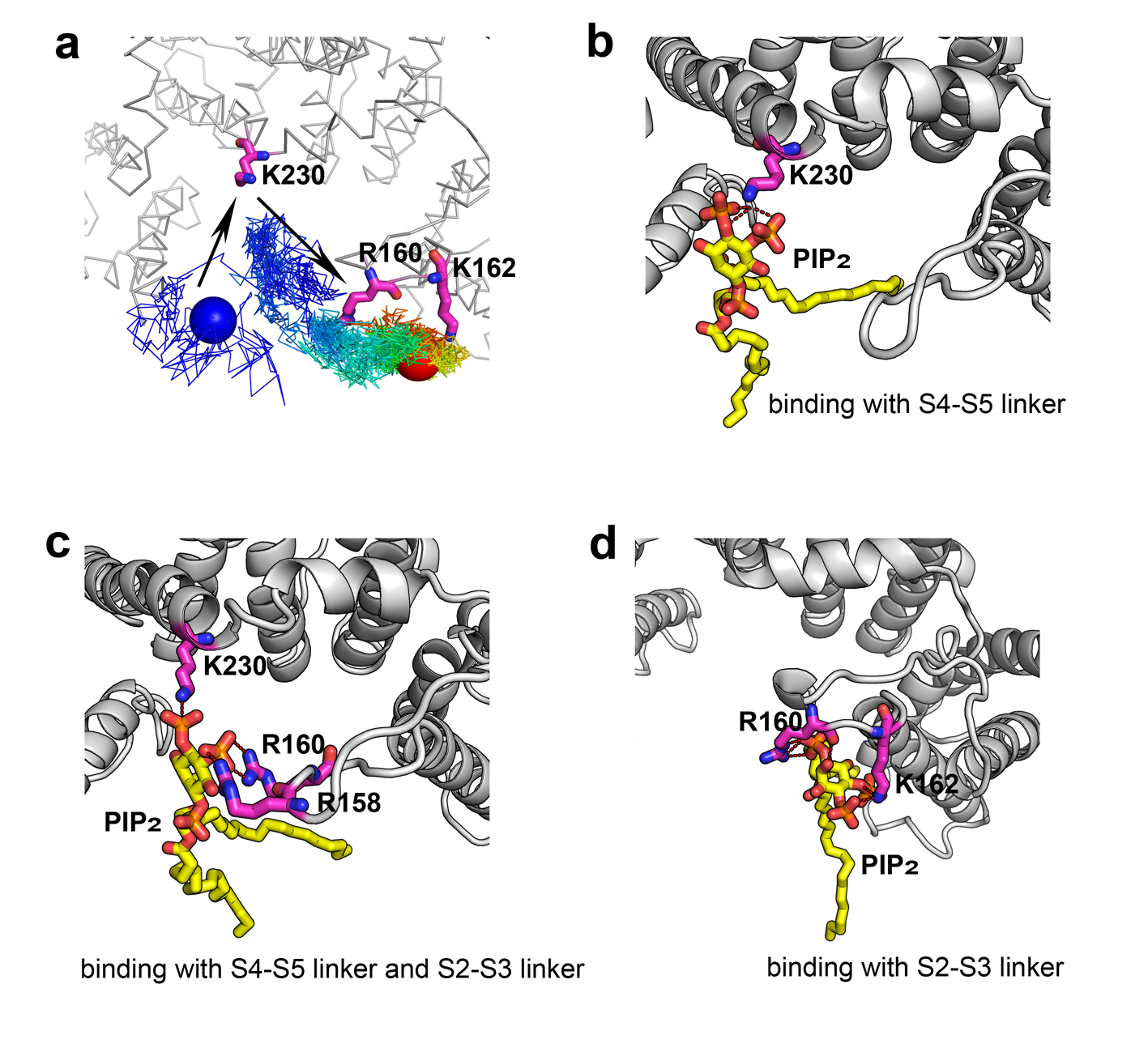
**

**Supplementary Figure 2.** Three typical binding models of PIP_2_ and KCNQ2 channel during the PIP_2_ migration process. (**a**) One of the migration trajectories of PIP_2_ molecule between S4-S5 linker and S2-S3 linker in the simulations of the open state KCNQ2 channel. (**b**, **c**, and **d**) Intracellular side view of the three representative binding models of PIP_2_ during the migration trajectory: (**b**) PIP_2_ binding with the S4-S5 linker, (**c**)PIP_2_ binding simultaneously with the S4-S5 linker and S2-S3 linker, and (**d**) PIP_2_ binding with the S2-S3 linker.

**
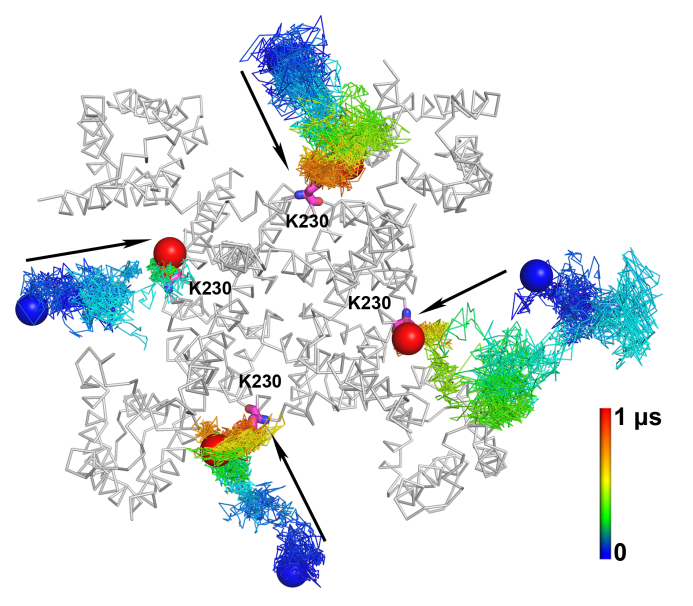
**

**Supplementary Figure 3.** Trajectories of PIP_2_ molecules in the simulation of the open-state KCNQ2 channel with neutral mutations of the S2-S3 linker. When all of the positive residues of the S2-S3 linker (R153, R155, R158, R160, K162, R165, and K166) are mutated to alanine (A), all four PIP_2_ molecules move to the S4-S5 linker. Display styles are the same as in Figure 1.


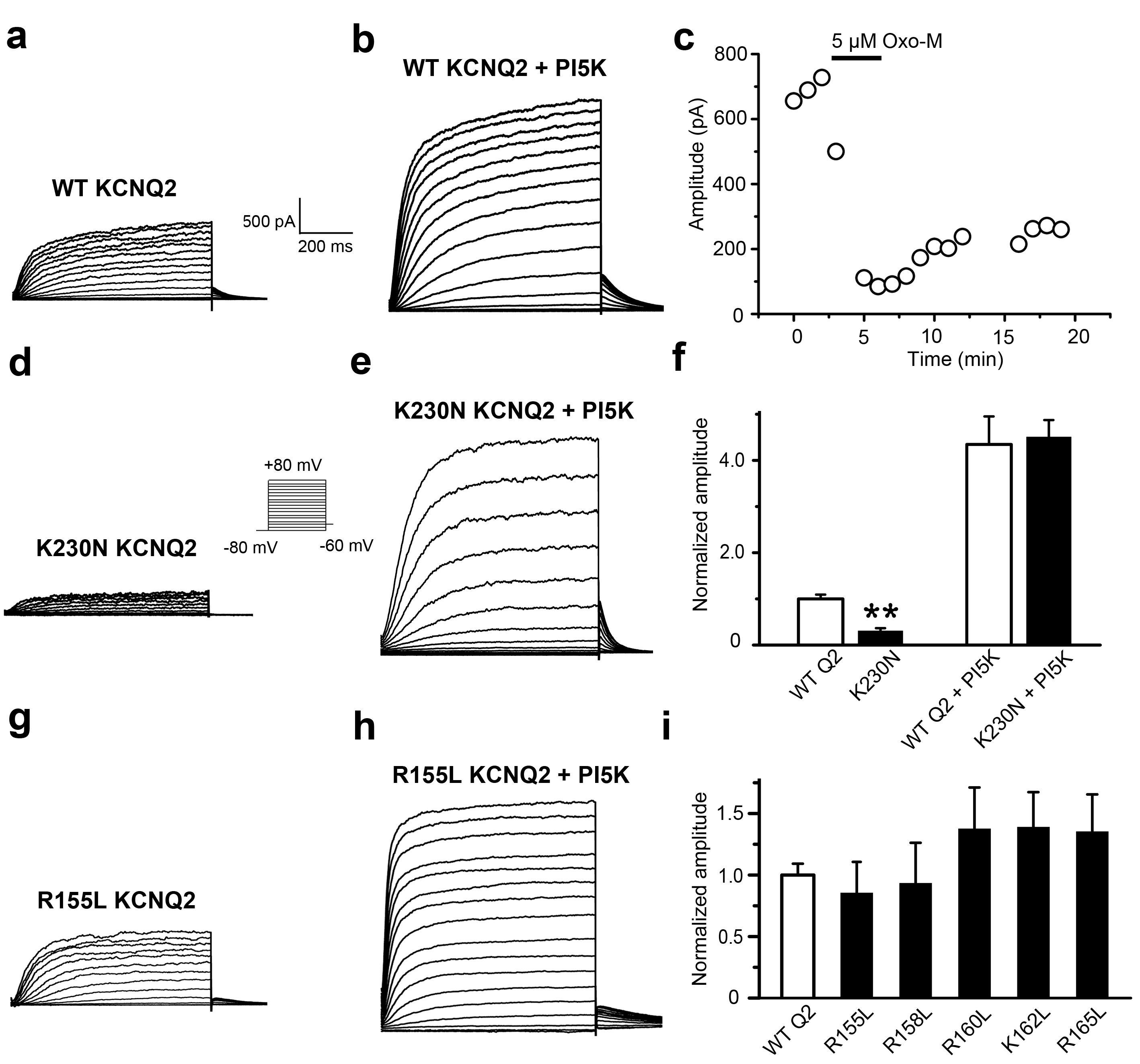


**Supplementary Figure 4.** The typical "M current" behaviors of the WT KCNQ2 channel and its mutants. (**a**,**b**) The typical current traces of WT KCNQ2 currents with or without PI(4)5-kinase. The holding potential depolarized from -80 mV to +80 mV in 10 mV steps, and then stepped down to -60 mV. (**c**) Activation of M1 receptor by Oxo-M mediated the inhibition of KCNQ currents. (**d**,**e**) The typical current traces of mutant K230N at the S4-S5 linker of KCNQ2 with or without PI(4)5-kinase. (**f**) Normalized current amplitudes of WT and K230N KCNQ2 channels with or without PI(4)5-kinase. (**g**,**h**) The typical current traces of mutation at S2-S3 linker of KCNQ2 channel with or without PI(4)5-kinase. (**i**) Normalized current amplitudes of WT KCNQ2 channel and its mutants (normalized to WT KCNQ2). The significant difference is indicated by paired t-test: **, P<0.01.

**
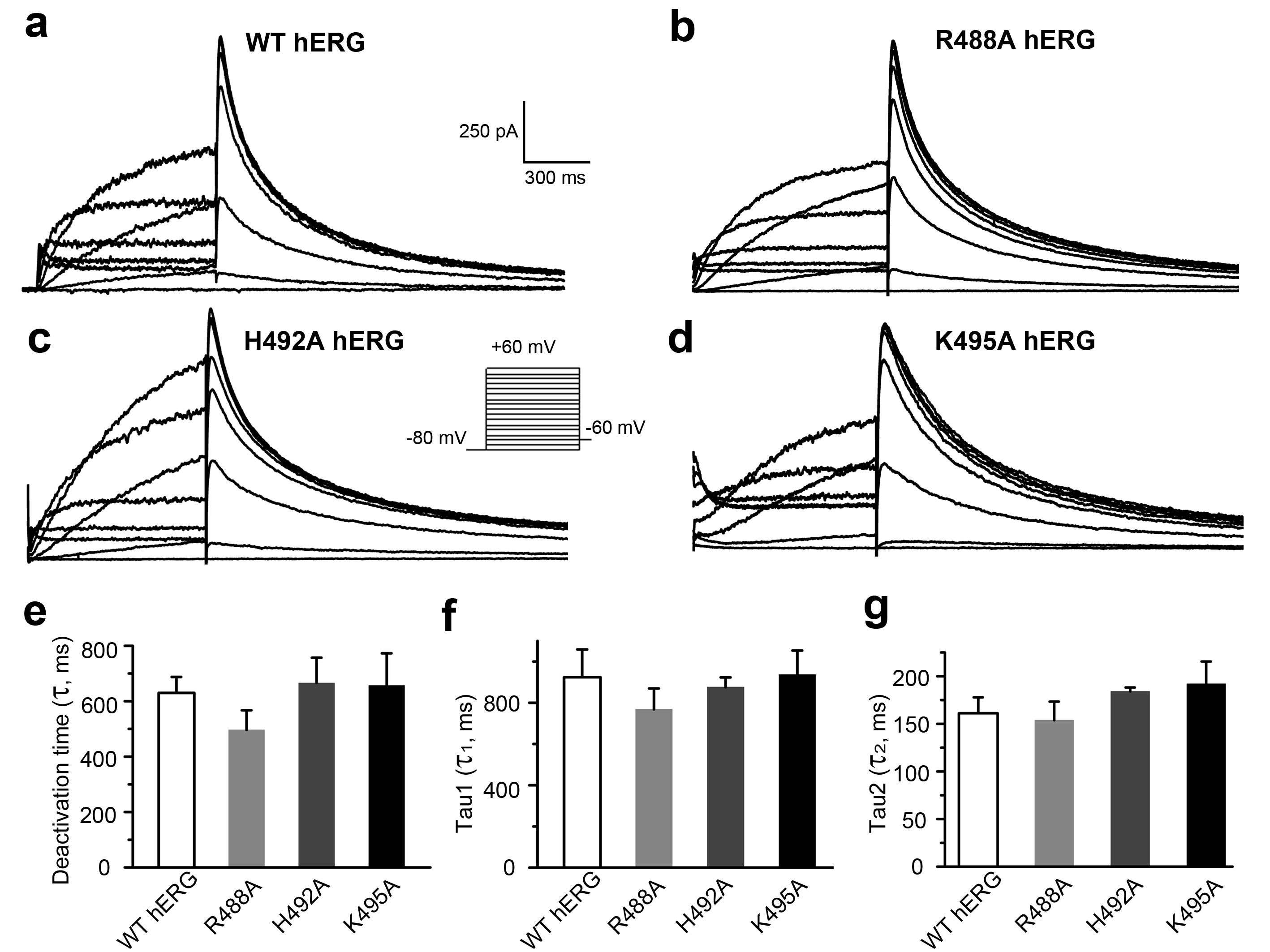
**

**Supplementary Figure 5.** The current traces and the deactivation time constants of WT and mutant hERG channels. (**a-d**) The typical current traces of WT (**a**) and mutations of hERG channels (**b**-**d**). The holding potential depolarized from -80 mV to +60 mV in 10 mV steps, and then stepped down to -60 mV. (**e**) The weighted deactivation time constant of WT and mutations of hERG channels according to the equation: τ_deact_ = (A_1_τ_1_ + A_2_τ_2_)/(A_1_ + A_2_) (see method). (**f,g**) The slow and fast deactivation time constants of WT hERG channel and its mutants. τ_1_ means the slow deactivation time constant and τ_2_ means the fast deactivation time constant.

**
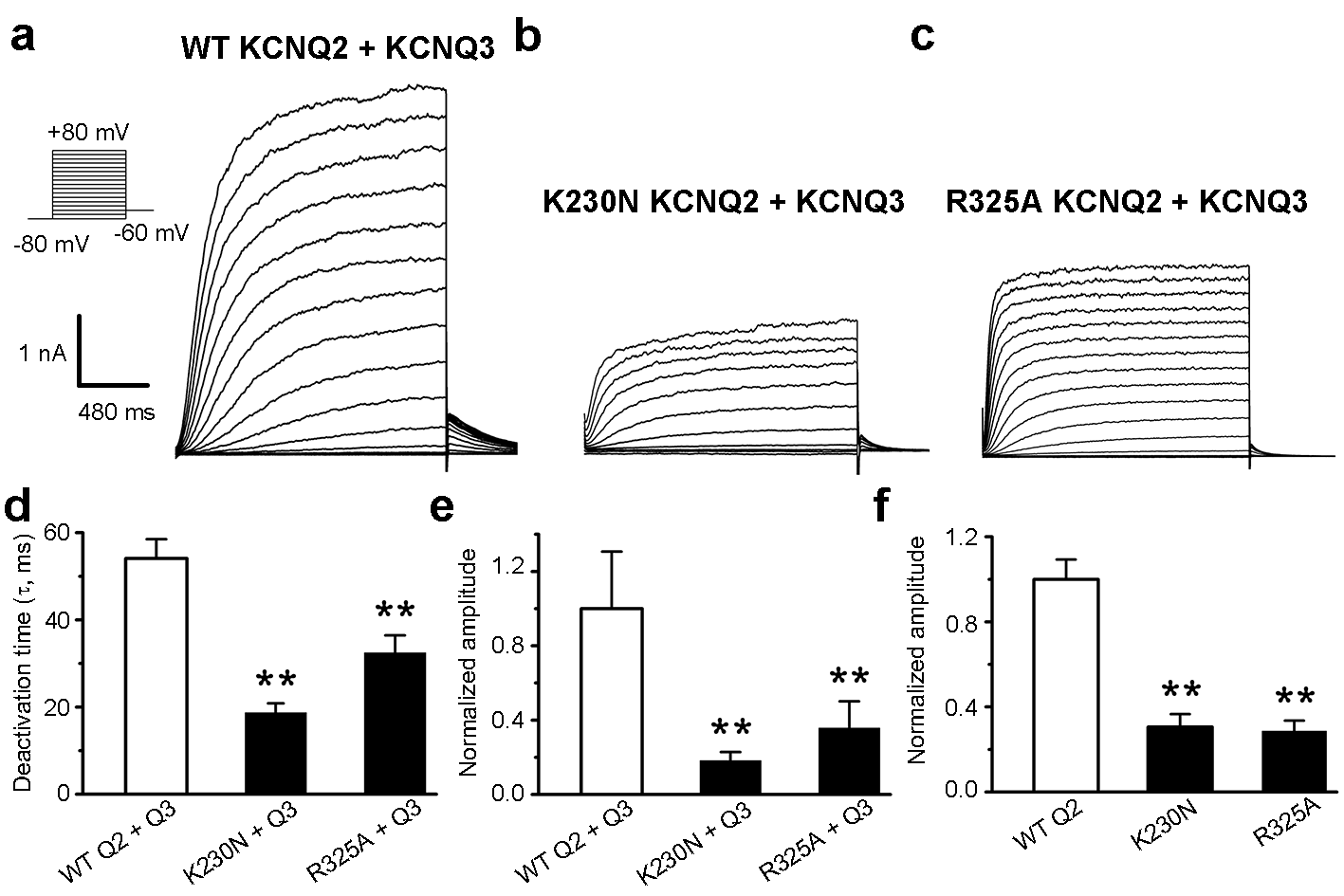
**

**Supplementary Figure 6.** Typical current traces and the behaviors of WT and mutated KCNQ2 channels co-expressed with KCNQ3 channel. (**a-c**) Current traces of WT and mutated KCNQ2 channels co-expression of the KCNQ3 channel. The holding potential was held at -80 mV, then depolarized to +80 mV in 10 mV increments and then stepped down to -60 mV. (**d**) The deactivation time constants of WT and mutated KCNQ2 channels co-expressed with the KCNQ3 channel. The deactivation time constants were obtained by an exponential fit of the tail currents. (**e**, **f**) Normalized current amplitudes of WT and mutated KCNQ2 channels with co-expression of the KCNQ3 channel (normalized to WT KCNQ2 co-expressed with the KCNQ3 channel). Data were shown as means ± SE. The significant difference is indicated by paired t-test: **, P<0.01.

**Supplementary Tables**

**Supplementary Table 1**. The percentage of time during simulations when PIP_2_ molecules stayed in the membrane, contacted with the S4-S5 linker, contacted with the S2-S3 linker, and simultaneously interacted with the S4-S5 linker and S2-S3 linker, for the WT KCNQ2 channel and the R160A mutant.

| Systems | | | Membrane | S4-S5 linker | S2-S3 linker | S4-S5 Linker + S2-S3 linker |
| --- | --- | --- | --- | --- | --- | --- |
| **/** | | Simulations |  |  |  |  |
| WT | 15 Å | 1 | 4% | 49% | 36% | 11% |
|  |  | 2 | 6% | 45% | 27% | 22% |
|  | 20 Å | 1 | 20% | 34% | 44% | 2% |
|  |  | 2 | 15% | 32% | 38% | 15% |
|  | average | | **11%** | **40%** | **36%** | **13%** |
| R160A | 15 Å | 1 | 8% | 70% | 20% | 2% |
|  |  | 2 | 9% | 50% | 32% | 9% |
|  | 20 Å | 1 | 14% | 62% | 24% | **/** |
|  |  | 2 | 14% | 83% | 3% | **/** |
|  | average | | **11%** | **66%** | **20%** | **3%** |

**Supplementary Table 2.** Deactivation time constants of the WT and mutant KCNQ2 channels.

| Construct | τ_deact_ (ms) | *n* |
| --- | --- | --- |
| WT KCNQ2 | 64.5 ± 5.3 | 18 |
| R155L | 116.5 ± 12.7* | 5 |
| R158L | 82.9 ± 4.0 | 7 |
| R160L | 107 ± 20* | 10 |
| K162L | 98 ± 5.8 | 7 |
| R165L | 120 ± 23* | 4 |
| WT KCNQ2 + PI5K | 77.1 ± 7.4 | 19 |
| K230N + PI5K | 15.3 ± 1.6* | 4 |
| WT KCNQ2 +KCNQ3 | 54.1 ± 4.4 | 8 |
| K230N +KCNQ3 | 18.6 ± 2.2* | 6 |
| R325A +KCNQ3 | 35.5 ± 3.0* | 7 |

*Note*: PI5K means PI(4)5-kinase. τ: deactivation time constant. *n* : the number of recorded cells. *** p < 0.05 *vs* WT**

**Supplementary Table 3. Summary of deactivation time constants of WT KCNQ1 channel and its mutants in S2-S3 linker.**

| Construct | τ_deact_ (ms) | *n* |
| --- | --- | --- |
| WT KCNQ1 | 596 ± 99 | 7 |
| W188R | 230 ± 33* | 7 |
| V185R | 314 ± 26* |  |

*Note*: τ means deactivation time constant. *n* means the number of recorded cells. *** p < 0.05 *vs* WT**

**Supplementary Table 4.** Summary of deactivation time constants of WT hERG and its mutants at S2-S3 linker.

| Construct | τ_1deact_ (ms) *^a^* | τ_2deact_ (ms) *^b^* | τ_deact_ (ms) | *n* |
| --- | --- | --- | --- | --- |
| WT | 923 ± 134 | 161 ± 16.6 | 630 ± 57 | 13 |
| R488A | 768 ± 101 | 154 ± 20 | 497 ± 70 | 8 |
| H492A | 876 ± 47 | 184 ± 4.2 | 666 ± 91 | 8 |
| K495A | 936 ± 116 | 192 ± 23 | 656 ± 116 | 13 |
| W497K | 366 ± 41 | 51 ± 2.4 | 135 ± 15* | 7 |

*Note*: *a*,τ_1_ is the slow deactivation time constant; *b*,τ_2_ is the fast deactivation time constant. *n* means the number of recorded cells. *** p < 0.05 *vs* WT**
